# State-level suicide mortality insights: a comparative study of VHA veterans and the whole US population

**DOI:** 10.1093/pubmed/fdaf036

**Published:** 2025-04-06

**Authors:** Viswadeep Lebakula, Angela R Cunningham, Arthur G Cosby, Anuj Kapadia, Jodie Trafton, Alina Peluso

**Affiliations:** Geospatial Science and Human Security Division, National Security Sciences Directorate, Oak Ridge National Laboratory, PO Box 2008, Mail Stop 6017, Oak Ridge, TN 37831, USA; Geospatial Science and Human Security Division, National Security Sciences Directorate, Oak Ridge National Laboratory, PO Box 2008, Mail Stop 6017, Oak Ridge, TN 37831, USA; Social Science Research Center, Mississippi State University, 1 Research Blvd, Starkville, MS 39762, USA; Computational Sciences and Engineering Division, Computing and Computational Sciences Directorate, Oak Ridge National Laboratory, PO Box 2008, Mail Stop 6017, Oak Ridge, TN 37831, USA; Office of Mental Health and Suicide Prevention, Veterans Health Administration, 3801 Miranda Avenue, Palo Alto, CA 94304, USA; Computational Sciences and Engineering Division, Computing and Computational Sciences Directorate, Oak Ridge National Laboratory, PO Box 2008, Mail Stop 6017, Oak Ridge, TN 37831, USA

**Keywords:** clustering, epidemiology, spatial analysis, suicide, US veterans

## Abstract

**Background:**

Suicide is a leading cause of death in the US Comparative State-level spatial analysis between Veterans Health Administration (VHA veterans) and the whole US population can reveal differences in conditions for targeted interventions and intricate geographical patterns.

**Methods:**

The study population contains 2018 and 2019 suicide deaths of VHA veterans and the whole US population. They were used to calculate state-level rates. States were classified by whether their VHA veteran and whole US population rates were above or below respective mean rates. Local Moran’s I was leveraged to examine spatial autocorrelation.

**Results:**

State-level suicide mortality rates and disparities among states were generally higher for VHA veterans (2018: 37.3 ± 7.2; 2019: 46.8 ± 8.3) than for the whole US population (2018: 16.6 ± 4.3; 2019: 16.4 ± 4.4). For both populations, there were statistically significant clusters with high suicide rates. Over one-fourth of states demonstrated inverse relationships, with rates above mean for one group but below for other. VHA veterans are at higher risk with over one-third of states had greater than average veteran suicide risk ratio.

**Conclusions:**

VHA veterans are at higher risk than the whole population across all states. Mortality disparities among states and clusters of states with high and low rates suggest targeted interventions and cooperative health strategies may help address these differences.

## Introduction

Suicide stands as a major public health issue, being one of the foremost causes of mortality globally.^1–3^ In the USA, suicide mortality consistently ranked as a leading cause of death,^4^^,^^5^ with suicide rates increasing by ~ 35% during 1999–2018, despite global and previous national declines between 1986 and 1999.^6–8^ There was a significant departure from this troubling trend in 2018–19, marked by a decline in suicides for the first time in over a decade.^5^^,^^7^ Another decline in suicide mortality followed in 2019–20 before the increasing trend resumed in 2020–21 time period. While the impact of COVID-19 on suicide mortality rates during 2019–20 remains uncertain and is not part of this analysis, the pandemic represents an unprecedented event in mortality studies, which could distort long-term trends and make it more difficult to interpret the underlying causes of observed changes. The 2018–19 period, by contrast, is more typical of the broader, prepandemic trajectory of suicide rates, providing a clearer understanding of the factors that may have contributed to the observed decline. So, in this work, we have focused on the 2018–19 period.

It is crucial to investigate suicide mortality at policy-relevant scales that include country, state, and county levels. Comparative analysis between the US veterans under Veterans Health Administration care^a^ (VHA veterans) and the whole population can provide insights into impact of military service on suicides. State-level spatial modeling has profound impact on public health interventions that include suicide prevention initiatives, support for at-risk individuals, and strategies to prevent reattempts.^9–15^ Comparative analysis between two populations at the county level on a yearly basis does not result in robust conclusions because suicide data is unreliable at the county level primarily due to high inter-annual variability and the rare outcome nature (Supplementary Table 1). To address this unreliability, researchers excluded counties with less than 10 or 20 deaths.^16^^,^^17^ This issue gets pronounced for VHA veterans with only less than 1% (2018: 0.5%; 2019: 0.9%) of counties have suicide deaths greater than 10.

There exists a dearth of comprehensive research comparing US whole population and VHA veterans^13–15^ with particularly scant attention devoted to state-level spatial comparisons during the recent period of decline. Addressing this gap, our research endeavors to analyze state-level spatial disparities in suicide mortality during 2018–19 for both populations.

## Methods

Suicide deaths for VHA veterans and the whole US population for 2018 and 2019 were obtained from the National Death Index and the National Vital Statistics System database, using ICD-10 codes U03, X60-X84, and Y87, covering 95 786 (48 312 in 2018 and 47 474 in 2019) suicide deaths, with 7000 (3492 in 2018 and 3508 in 2019) being VHA veterans under VHA care. The empirical Bayes approach was used to calculate state-level smoothed suicide rates (EB rates) per 100 000.^2^^,^^18^^,^^19^ It is preferred over standardized mortality rates (SMR) as SMR can over-interpret small change in deaths as a large variation.^20^ This variation issue can further propagate into clustering, which results in misidentification of clusters.

We conducted cluster analysis using Anselin Local Moran’s I on EB rates.^21^ Standard deviation (SD) was used as a measure of disparities in mortality rates among states. States were categorized into four groups: greater than mean rates for both populations, greater than mean rate for the whole population only, greater than mean rate for VHA veterans only, and lower than the mean rates for both. VHA veteran suicide risk ratio was calculated as VHA veteran rates over the whole population rates.

## Results

**Figure 1:**
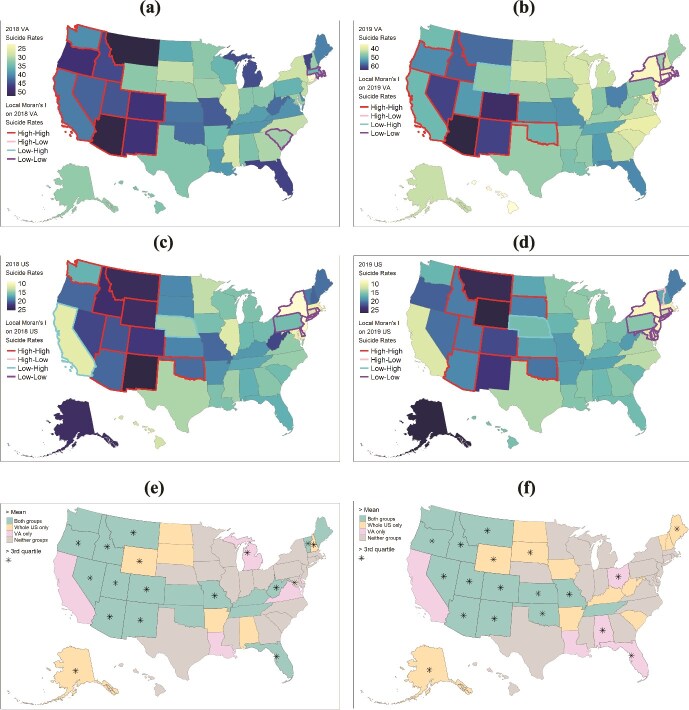
State-level distributions of suicide mortality for US VHA veterans and the whole population from 2018 to 2019. Maps of suicide rates along with clusters from local Moran’s I for US VHA veterans in 2018 (a) and 2019 (b) and the whole US population in 2018 (c) and 2019 (d). *High-high* and *low-low* indicate clusters of high and low suicide rates, respectively. *High-low* and *low-high* identify outlier states with high rates surrounded by states with low rates and with low rates surrounded by states with high rates, respectively. Categorical maps of suicide mortality greater than the mean suicide rates for VHA veterans and the whole US population combined in 2018 (e) and 2019 (f). For each year, mean rates were calculated separately for the two populations. Based on these mean rates, we divided all states into four categories that include higher than mean rate for both populations (green), higher than mean for the whole population only (yellow), higher than mean for VHA veterans only (pink), and lower than mean rate for both populations (gray). States with suicide mortality rates higher than the third quartile are marked with a star.

Suicide rates and the disparities (SD) among rates were higher for the VHA veterans than the whole population in both 2018 and 2019 (Fig. 1a–d). In 2019, the state rates shifted toward higher rates for the VHA veterans (mean increased from 37.3 to 46.8) but not for the whole population (mean remained at ~16). The SD of rates among states were 7.2 for VHA veterans in 2018 and 4.3 for the whole population. In 2019, SD values increased to 8.3 for VHA veterans and 4.4 for the whole population.

Local Moran’s I clustering on yearly suicide rates suggests that there were statistically significant clusters of high rates for both populations in the West (Fig. 1a–d). A persistent lower mortality cluster was observed in the northeast for the whole population. For the whole population, Nebraska was the only persistent outlier, having a low suicide rate surrounded by states with high rates.

The number of states with rates greater than mean varied across four categories (Fig. 1e and f). Most states had rates either above (2018: 35.3% and 2019: 25.5%) or below (2018: 39.2%; 2019: 39.2%) the mean rate for both populations. 13.7% (2018) and 23.5% (2019) of states had suicide rates above the mean for the whole population but below the mean for VHA veterans. Whereas in both years only 11.8% of states had rates above the mean for VHA veterans but not for the whole population.

**Figure 2:**
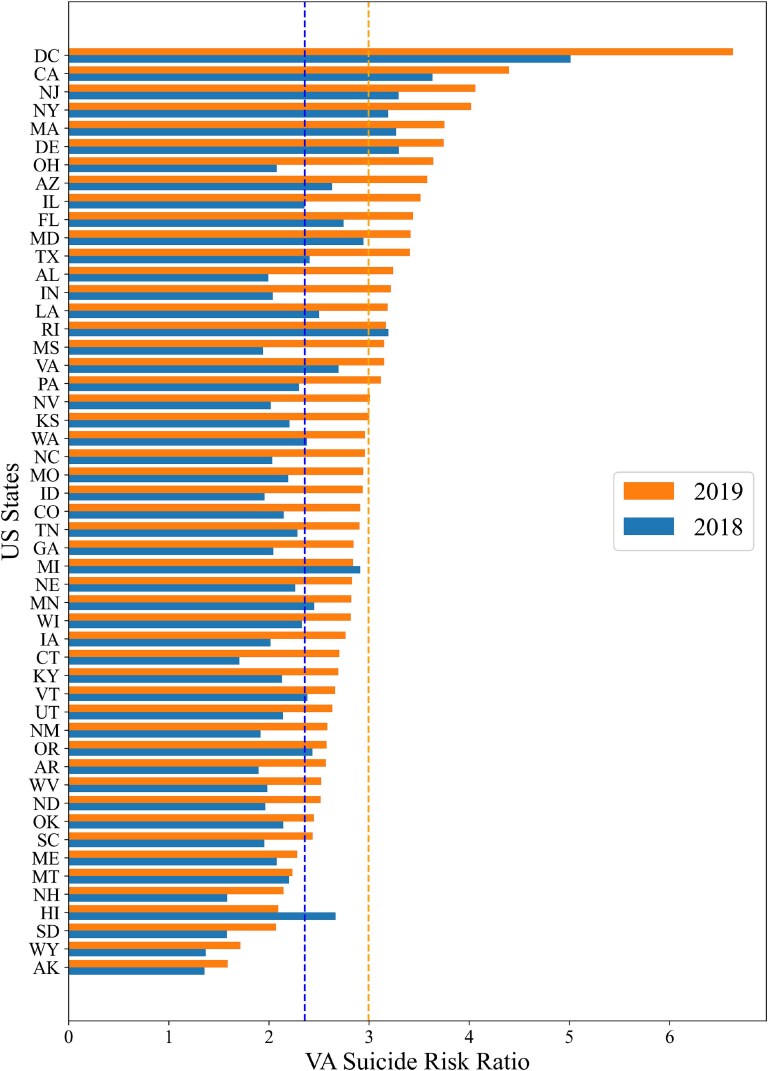
State-level relative risk for VHA suicide in contrast to the whole population within US States for 2018 and 2019 (c). The blue and orange lines represent mean VHA veteran suicide risk ratio for 2018 and 2019.

While state-level VHA Risk Ratio estimates varied among states, in every state there was a substantially higher suicide risk for that state’s VHA veteran population (Fig. 2), and this pattern existed in both years. Comparing 2018’s VHA Risk ratio to 2019’s revealed an additional pattern was observed. There was a strong tendency for VHA Risk Ratio to increase from 2018 to 2019. Out of 50 states and the District of Columbia, relative VHA Risk Ratio increased in all but four states.

### Main finding of this study

For 2018 and 2019, statistically significant clusters of states with higher mortality rates were observed in the Western US for VHA veterans and whole US populations. Between these two groups, across all the states, the VHA veterans had substantially elevated suicide risk with higher suicide mortality rates. Also, the disparities of rates among states were higher for the VHA veterans. Additionally, a comparative analysis of VHA Risk Ratios between 2018 and 2019 revealed a clear upward trend, with the relative VHA Risk Ratio increasing in 46 out of 50 states and the District of Columbia.

### What is already known on this topic

Previously researchers have compared VHA veteran population to the general US population. This research has consistently demonstrated that the veteran population has higher rates than the whole US population.^14^ Among veterans, VHA vetran suicide rates were lower than veterans who are not under VHA care.^15^ Gender and age have been shown to be important factors underlying veteran suicide rates. In addition, research utilizing controls for age and gender have shown that the number of veteran suicides has increased substantially during recent years. This growth pattern also included an elevated suicide risk for female veterans. These trends occurred in both the veterans who utilized VHA and those who did not utilized VHA services.

### What this study adds

In this study, we have extended the analysis to include substantial geographic context of suicide rates between the VHA veterans and the whole US population at the state level. We estimates state-level risk for both populations, which provides more granular measures of suicide disparities at the state level. The geographic analysis also was further supplemented by spatial statistical analysis to identify clusters of similar suicide patterns.

### Study limitations

As with many research endeavors, this study generates many questions for future analysis. First, we report that the elevated suicide risk for VHA veterans is pervasive across states. This finding however is based only on 2 years (2018 and 2019). Analysis across a larger time frame needs to be conducted to have a better idea of the temporal persistence of this high-risk population. Second, we can leverage the considerable variation in suicide data between states to model and predict suicide outcomes based on the various characteristics of states. This strategy is often used to understand all cause and disease mortality. Third, there are some interesting outliers that possibly could produce usable information produced by case studies. The District of Columbia data had very high-risk factors for VA veteran suicide and a very low risk for the general district population. Likewise, a more promising case study would be a study of the New England cluster, which has the most favorable data for suicide prevention. In designing each of these studies, a priority should be to include variables/influences that are either malleable or predictive in improving suicide outcomes. Fourth, we did not account for discrepancies in reported suicide deaths at the state level, which have been previously identified by researchers.^22^^,^^23^ For instance, states that rely solely on coroners for death investigations have been found to underreport suicides compared to states with medical examiners.^22^^,^^23^ These reporting differences may impact the accuracy of suicide mortality data and should be considered when interpreting the findings.

## Conclusions

American life expectancy differs from other nations and varies significantly within the country at regional, state, and county levels. This research focuses on state-level patterns and comparison between VHA veterans and whole population rates, and how these rates change during 2018–19. This analysis reveals patterns in state-specific suicides that can inform targeted interventions, collaborative responses, and future research priorities.

National data has consistently revealed that VA suicide rates significantly exceed whole population rates. This study demonstrates that this pattern of higher VA suicide rates is pervasive across all state boundaries. Within each state, the VHA veteran rates were substantially higher than the whole population. This relative VHA risk pattern was consistent, with VHA risk exceeded twice of the whole population and thrice in several states. From a public health policy perspective, the pervasiveness and consistency of greater VHA risk is strong evidence that VA suicide should be both a national and a state-level policy issue. Also, the increased VHA risk during 2018–2019 was primarily due to a decline in rates for the whole population but not VHA veterans. This raises a question, why should VHA veteran rates remain high while general rates were declining?

Cluster analysis identifies two distinct clusters: one in the western US with high suicide rates for both VA and the whole population, and another in New England with low rates for both groups. This outcome suggests that the western cluster may benefit from increased collaboration and collective health strategies to address high rates, while the northeastern cluster’s protective environment from suicide could provide insights into transferable factors to enhance prevention efforts nationwide.

## Supplementary Material

Supplementary_Information_fdaf036

## Data Availability

For this study we used suicide mortality data for the VHA veterans and the whole U.S. population. They are available to download from from the National Death Index (https://www.cdc.gov/nchs/ndi/index.html) and the National Vital Statistics System database (https://www.cdc.gov/nchs/nvss/index.htm).

## Notes

**V. Lebakula,** Research Scientist

**A. R. Cunningham,** Spatial Demographer

**A. G. Cosby,** Distinguished Professor Emeritus

**A. Kapadia,** Distinguished R&D Scientist

**J. Trafton,** Director, Program Evaluation and Resource Center

**A. Peluso,** Research Scientist
