# Supplementary material for: State-level suicide mortality insights: a comparative study of VHA veterans and the whole US population
Source: J Public Health (Oxf). 2025 Apr 6;47(2):188–93. doi: 10.1093/pubmed/fdaf036 (PMC12123301; doi:10.1093/pubmed/fdaf036)
Supplement: Supplementary_Information_fdaf036 [file supplementary_information_fdaf036.docx]

Supplementary Information

Supplementary Table I: Counties with Suicide Counts for the VHA veterans and the Whole U.S. Population: 2018-2019

| Counties with suicide counts | Year | VHA Veterans | Whole U.S. |
| --- | --- | --- | --- |
| = 0 | 2018 | 2268 | 341 |
| >10 | 2018 | 16 | 940 |
| >20 | 2018 | 4 | 534 |
| =0 | 2019 | 2125 | 331 |
| >10 | 2019 | 28 | 906 |
| >20 | 2019 | 4 | 517 |

Note: Among 3,143 U.S. counties, for the veteran population, in 2018, there were only 16 and 4 counties that have suicide deaths greater than 10 and 20. For the veteran population, in 2019, there were only 28 and 4 counties that have suicide deaths greater than 10 and 20. For the whole U.S. population, in 2018 there were 940 and 534 counties that have suicide deaths greater than 10 and 20. For the whole U.S. population, in 2019 there were 906 and 517 counties that have suicide deaths greater than 10 and 20.
